# Salivary Alpha-Amylase and Psychosocial Factors in Acute and Chronic Orofacial Pain—An Observational Cross-Sectional Study

**DOI:** 10.3390/dj14100688

**Published:** 2026-10-21

**Authors:** Maibel Attaalla, Nikolaos Christidis, Hajer Jasim

**Affiliations:** 1 Division of Oral Diagnostics and Rehabilitation, Department of Dental Medicine, Karolinska Institutet, SE-14104 Huddinge, Sweden; 2 Folktandvården Flen, Public Dental Services, Folktandvården Sörmland AB, SE-642 35 Eskilstuna, Sweden; 3 Department of Orofacial Pain and Jaw Function, Eastman Institutet, Public Dental Services, Folktandvården Stockholm AB, SE-102 31 Stockholm, Sweden

**Keywords:** orofacial pain, salivary alpha-amylase, temporomandibular disorders, acute pain, chronic pain, psychological

## Abstract

**Background:** Orofacial pain (OFP) is a common clinical problem. It may present as acute or chronic pain and involves different biological and psychosocial factors. Acute OFP is often related to odontogenic causes and short-term nociceptive activation, whereas chronic OFP, particularly temporomandibular disorder (TMD), is frequently associated with stress, depression, and other psychosocial symptoms. Salivary alpha-amylase (sAA) reflects autonomic nervous system activity and has been suggested as a non-invasive biomarker of stress-related pain. However, its role in OFP and its relation to psychosocial burden remains unclear. **Objectives:** To investigate sAA activity and psychosocial factors in women with acute and chronic OFP compared with pain-free controls. **Methods:** A total of 144 women were included: 47 with acute OFP, 47 with chronic OFP diagnosed according to diagnostic criteria for TMD, and 50 pain-free controls. Psychological variables were assessed using the Perceived Stress Scale (PSS-14) and the Symptom Checklist-90-Revised short version (SCL-32-R). Unstimulated saliva samples were collected and analysed for sAA activity using a kinetic enzyme assay. **Results:** Significant group differences in sAA activity were observed (*p* = 0.038). Patients with acute OFP showed higher sAA activity compared with controls (*p* < 0.05), whereas no significant differences were found between chronic OFP and controls. Across all participants, a weak positive pooled correlation was observed between sAA activity and current pain intensity (r_s_ = 0.23, *p* < 0.007). Chronic OFP patients reported higher levels of non-specific physical symptoms and current stress compared with controls (*p* < 0.01). Within the chronic OFP group, depressive symptoms, perceived stress, and non-specific physical symptoms were strongly correlated. **Conclusions:** Acute and chronic OFP were associated with different patterns: acute OFP was associated with higher sAA activity than in controls, whereas chronic OFP was associated with higher current stress and non-specific physical symptoms, but not with higher sAA activity. These cross-sectional findings should be interpreted cautiously and do not establish that the observed differences are attributable to pain duration alone.

## 1. Introduction

Orofacial pain (OFP) is a common condition and one of the leading causes of emergency and specialist dental visits. OFP encompasses a heterogeneous group of pain conditions affecting the face, mouth, and jaws and can be classified according to its anatomical origin, underlying mechanism, and temporal pattern [1]. Depending on duration and underlying mechanisms, OFP may present as acute or chronic pain. Acute OFP is often odontogenic and associated with ongoing tissue injury or inflammation, whereas chronic OFP, particularly temporomandibular disorders (TMD), is associated with more complex biological and psychosocial processes [2,3].

Acute pain generally serves as a protective function and is closely related to ongoing peripheral nociceptive input. By contrast, chronic pain may persist beyond the expected period of tissue healing and can involve peripheral and central sensitization, altered pain modulation, comorbid pain conditions, and psychosocial influences [4]. Importantly, odontogenic pain and TMD differ not only in pain duration but also in anatomical origin, pathophysiology, inflammatory burden, and clinical picture. Comparisons between these conditions should therefore not be interpreted as reflecting pain chronicity alone.

TMD are among the most common chronic pain conditions affecting the face and jaw. They include pain-related and intra-articular conditions involving the masticatory muscles, temporomandibular joints, and associated structures and may result in pain, impaired jaw function, and pain-related disability [5]. The etiology is considered multifactorial and may involve behavioral, psychological, and psychosocial factors in addition to peripheral nociceptive input [6,7]. Accordingly, the Diagnostic Criteria for TMD (DC/TMD) integrate clinical diagnoses with assessment of pain-related disability, psychological distress, and functional impact [5]. Previous studies have shown associations between chronic TMD pain and stress, depressive symptoms, anxiety, and non-specific physical symptoms [8,9,10,11]. Findings from the OPPERA study further indicated that somatic symptoms, perceived stress, negative affectivity, and stressful life events may precede the onset of painful TMD, although the strengths of these associations differed across psychosocial domains [11]. More recent clinical studies comparing acute and chronic painful TMD have reported greater pain-related interference, disability, somatic symptom burden, depression, and anxiety in patients with chronic TMD, while also demonstrating that these factors are interrelated and cannot be regarded as uniform or independent features of all patients with persistent TMD pain [12,13]. These findings support a biopsychosocial conceptualization of chronic TMD in which biological, behavioral, and psychosocial factors may interact, rather than a model in which persistent pain is explained by peripheral nociceptive input alone.

The autonomic nervous system (ANS) and hypothalamic–pituitary–adrenal (HPA) axis play important roles in stress regulation and pain processing. Salivary alpha-amylase (sAA), an enzyme secreted mainly by the parotid glands, has been proposed as a non-invasive biomarker of sympathetic nervous system activity [14,15,16]. Its secretion is modulated by autonomic innervation of the salivary glands, including adrenergic signaling, and experimental stress studies provide support for an association between sAA reactivity and adrenergic activation [14,15,16,17]. Unlike cortisol, which reflects slower HPA-axis responses, sAA responds rapidly to psychological and physiological stressors and may therefore reflect short-term autonomic activation [18,19]. However, sAA secretion can also be influenced by parasympathetic activity, physiological factors, salivary flow, age, circadian rhythm, collection procedure and handling [20,21,22].

Several studies have investigated sAA activity in stress-related and painful conditions, including TMD, although findings remain inconsistent. Experimental studies have demonstrated rapid sAA responses to psychosocial stress and pain, but the magnitude and direction of these responses have varied across pain models and study populations [18,23,24,25,26]. Clinical studies of acute dental pain have also produced differing results. Positive associations between pain intensity and sAA have been reported in patients with symptomatic irreversible pulpitis [27], during emergency endodontic treatment [28], and following mandibular third-molar surgery [24]. In contrast, smaller studies of dental extraction and experimental muscle pain found no association between sAA and pain intensity [26,29].

The evidence from chronic pain and TMD populations is similarly heterogeneous. Some studies have reported associations between sAA activity and pain intensity in chronic pain conditions [25,30], while others have found no significant associations [31,32]. Overall, these inconsistent findings suggest that sAA may be sensitive to acute nociceptive, emotional, or physiological challenges, but its relationship with persistent pain remains unclear [23,25]. Further studies are therefore needed to clarify whether sAA activity differs between acute and chronic OFP conditions.

Despite increasing interest in salivary biomarkers, few studies have compared acute and chronic OFP within the same methodological framework. Most previous studies have focused on either experimental pain, acute dental procedures, or chronic pain. To the best of our knowledge, no previous study has directly compared sAA activity between acute and chronic pain within the same methodological farmwork while also assessing psychosocial characteristics. In addition, the relationship between autonomic activation and psychosocial factors across different pain conditions remains insufficiently understood. A better understanding of these mechanisms may contribute to improved knowledge regarding biological and psychological processes involved in OFP [14,18,26].

The present study aimed to investigate sAA activity and psychosocial factors in women with acute and chronic OFP compared with pain-free controls. The study was designed as a cross-sectional comparison of clinically distinct OFP groups rather than as an investigation of temporal changes from acute to chronic pain. It was hypothesized that acute and chronic OFP would be associated with increased sAA activity compared with controls. Chronic OFP was also expected to be characterized by higher levels of psychosocial factors such as perceived stress, depressive symptoms, and non-specific physical symptoms.

## 2. Materials and Methods

### 2.1. Participants

This observational cross-sectional study included a total of 144 women aged 18–65 years were included in the study. Participants were divided into three groups: acute OFP (*n* = 47), chronic OFP (*n* = 47), and pain-free controls (*n* = 50). Group allocation was based on clinical status.

The general inclusion criteria were female sex, age 18–65 years, sufficient proficiency in Swedish to complete the questionnaire, and verbal and written informed consent. Patients with acute OFP presented with odontogenic pain of less than 10 days’ duration. The acute odontogenic pain group comprised patients with a range of odontogenic conditions, including symptomatic pulpitis, symptomatic apical periodontitis, acute apical abscess, and postoperative pain. Diagnosis was established by board certified dentists. Chronic OFP patients were diagnosed with painful TMD according to the Diagnostic Criteria for Temporomandibular Disorders (DC/TMD) [5] and reported pain duration exceeding 6 months. The DC/TMD examinations were performed by calibrated examiners. Controls consisted of pain-free individuals attending routine dental examinations with no ongoing or recurrent OFP. The absence of OFP in controls was confirmed by interview/anamnesis. Exclusion criteria included fibromyalgia, generalized pain conditions, neuropathic pain, systemic inflammatory disease, major systemic illness, pregnancy, and insufficient proficiency in Swedish. Only women were included to minimize potential confounding related to sex-related differences in pain perception [33], stress responses, and/or autonomic activity. Recruitment started in 2017, when 15 participants were enrolled. Following an interruption that was partly related to disruptions associated with the COVID-19 pandemic, recruitment resumed, with the majority of participants enrolled during 2023–2024. On each recruitment day, all patients attending the participating clinics who met the eligibility criteria were invited to participate, resulting in consecutive recruitment during these periods. Participants were recruited from general and specialist clinics in orofacial pain at the Eastman Institute, Folktandvården Stockholm, University Dental Clinic, Karolinska Institutet, Huddinge, and Folktandvården Flen, Sweden.

The study was approved by the Regional Ethical Review Board in Stockholm (2016/1964-31/3) and conducted in accordance with the Declaration of Helsinki. All participants provided written informed consent before inclusion.

### 2.2. Questionnaires and Clinical Variables

Pain intensities were assessed using a 0–10 numeric rating scale (NRS), where 0 represented no pain and 10 the worst imaginable pain. Perceived general health and oral health were each rated on a five-point scale, where 1 indicated excellent health and 5 indicated poor health.

Depressive symptoms and non-specific physical symptoms were assessed using the 32-item subset of the Symptom Checklist-90-Revised (SCL-90-R) included in the RDC/TMD questionnaire. The scale comprises 20 items assessing depressive symptoms and 12 items assessing non-specific physical symptoms. Each item is rated on a five-point scale from 0 to 4. Subscale scores are calculated by summing the item scores and dividing the total by the number of items in the corresponding subscale, yielding a mean score ranging from 0 to 4. Higher scores indicate greater symptom severity.

Perceived stress was assessed using the Perceived Stress Scale (PSS-14) [34]. The PSS-14 comprises 14 items rated from 0 (never) to 4 (very often). The seven positively worded items were reverse-scored, and all item scores were summed, producing a total score ranging from 0 to 56, with higher scores indicating greater perceived stress. Current stress was additionally rated using an NRS ranging from 0 to 10, where 0 represented no stress and 10 the worst imaginable stress. All questionnaires were completed at the clinic and reviewed by the clinician after completion. If some questions were left unanswered, the participants were asked to complete them.

Information regarding medication use, oral contraceptives, hormone therapy, smoking habits, and analgesic intake was collected through questionnaires and interviews.

### 2.3. Saliva Collection and Biochemical Analysis

Unstimulated whole saliva was collected according to standardized procedures. Most samples were collected between 09:00 and 14:00 during routine clinical activity to reduce circadian variation. The sampling schedule was intended to limit, although not eliminate, potential diurnal variation in sAA activity. The exact sampling time was recorded for each participant and compared across the three study groups.

Participants were instructed to refrain from eating, drinking, smoking, and oral hygiene procedures for at least 30 min before sampling.

Saliva was collected by passive drooling into sterile polypropylene tubes while participants remained seated in a relaxed position. Salivary flow rate was not measured. Samples were frozen at −20 °C within three hours after collection and stored until analysis. The samples were stored for maximum 18 months before analysis and underwent no repeated freeze–thaw cycles.

Before analysis, samples were thawed, vortexed, and centrifuged at 1500× *g* for 15 min to remove debris and mucins. sAA activity was analyzed using a commercially available kinetic enzyme assay kit (Salimetrics, State College, PA, USA) according to the manufacturer’s instructions. The intra-assay coefficient of variation ranged from 3% to 7%, and the inter-assay coefficient of variation ranged from 4% to 6%. All samples were analyzed in duplicate, and the mean of the duplicate measurements was used for statistical analysis.

### 2.4. Statistical Analysis

A priori power analysis was performed using G*Power version 3.1.9.7 (Düsseldorf, Germany). Pilot data indicated an overall effect size of Cohen’s *f* = 0.37 for differences in sAA activity among the three study groups. To account for the uncertainty associated with the relatively small pilot sample, a more conservative effect size of *f* = 0.30 was used for the sample-size calculation. Assuming a fixed-effects omnibus one-way ANOVA with three groups, a significance level of α = 0.05, and 80% power, a total sample size of 111 participants, corresponding to 37 participants per group, was required.

Normality was assessed using the Shapiro–Wilk test and visual inspection of histograms. As most variables were non-normally distributed, group differences were analysed using the Kruskal–Wallis test, followed by post hoc multiple comparisons of mean ranks using *z*-statistics, with Bonferroni adjustment for the three pairwise comparisons.

Association between sAA activity and clinical and psychosocial variables were examined using Spearman’s rank correlation. Associations between sAA activity and current pain intensity were examined separately within the acute and chronic OFP groups. Pain-free controls were excluded from this analysis because their current pain scores were zero. Bonferroni correction for multiple comparisons was applied. Sampling time was converted to minutes after midnight and compared among the study groups using the Kruskal–Wallis test. Sampling time data were available for 111 participants; for the remaining 33 participants, the exact sampling time was not documented in the study records. The analysis of sampling time was therefore based on the available cases (*n* = 111). Descriptive data are presented as mean ± standard deviation (SD) for normally distributed or continuous variables, median with interquartile range (IQR) for non-normally distributed variables, or number (%), as appropriate. For all analysis the significance levels were set to *p* < 0.05, except when an adjusted threshold was applied for multiple comparisons. Statistical analyses were performed using Statistica software version 14.2.0.18 (TIBCO Software Inc., Palo Alto, CA, USA).

## 3. Results

### 3.1. Participant Characteristics

A total of 144 women were included in the study, comprising 47 patients with acute OFP, 47 patients with chronic OFP, and 50 pain-free controls. Participant characteristics are presented in Table 1. Despite attempts to age-match the groups, age differed significantly between groups (*p* = 0.024), with patients in the chronic OFP group being slightly younger than those in the acute OFP group. Controls did not differ significantly in age from either patient group. Sampling did not differ significantly between groups, with median sampling time around 11:30 a.m. ((2,*N* = 111) = 3.56, *p* = 0.168).

Sociodemographic characteristics, including marital status, educational level, and employment status are presented in Table 1.

### 3.2. sAA Activity

**Figure 1:**
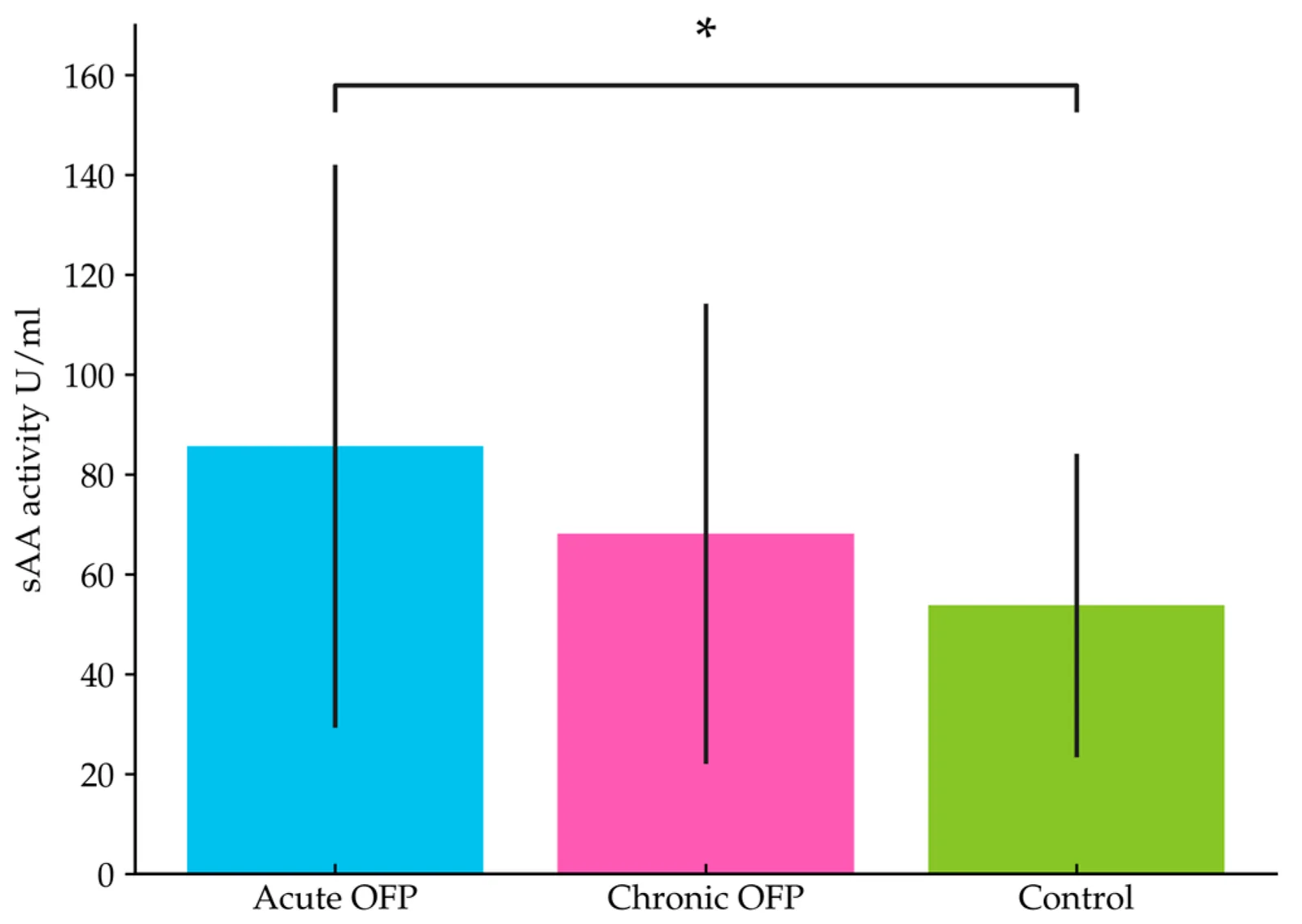
Salivary alpha-amylase (sAA) activity (U/mL) in 144 women with acute orofacial pain (OFP), chronic OFP, and pain-free controls. Bars represent mean values, and error bars indicate standard deviations (SDs). A significant group difference was observed (*p* = 0.038), with significantly higher sAA levels in patients with acute OFP (85.1 ± 56.3 U/mL) compared to controls (54.1 ± 30.3 U/mL). * *p* < 0.05 for the pairwise comparison between the acute OFP and pain-free control group.

A significant overall group difference was found for sAA activity (*H*(2, *N* = 144) = 6.54, *p* = 0.038). Patients with acute OFP demonstrated the highest sAA activity (85.1 ± 56.3; 78.7 (85.5) U/mL), which was significantly greater than that observed in controls (54.1 ± 30.3; 46.3 (37.1) U/mL) (*z* = 2.56, *p* < 0.05). The chronic OFP group showed intermediate sAA activity (68.5 ± 46.0; 577 (67.1) U/mL), which no significant differences compared with either the acute OFP group or controls (Figure 1).

### 3.3. Pain Characteristics and Analgesic Use

Pain intensity and analgesic use are presented in Table 2. Current pain intensity was significantly higher among participants with acute OFP (7 (3)) than among participants with chronic OFP (5 (3)); Mann–Whitney *U* = 531.0, adjusted *Z* = 3.55, *p* < 0.001, *r* = 0.38).

Pain duration was significantly longer among participants with chronic OFP than among those with acute OFP *H*(2, *N* = 144) = 132.84, *p* < 0.001). However, this difference was expected because pain duration formed part of the eligibility criteria used to define the acute and chronic OFP groups. Descriptively daily analgesic intake was reported by 26% of acute OFP patients, 9% of chronic OFP patients, and 2% of controls. Paracetamol and non-steroidal anti-inflammatory drugs were the most frequently used medications.

### 3.4. Psychological Variables

Significant group differences were found for non-specific physical symptoms (*H*(2, *N* = 144) = 24.17, *p* < 0.001). Chronic OFP patients reported the highest symptom levels and differed significantly from both acute OFP patients and controls. Acute OFP patients did not differ significantly from controls.

Self-rated current stress also differed significantly between groups (*H*(2, *N* = 144) = 9.33, *p* = 0.009). Chronic OFP patients reported significantly higher stress levels than controls, whereas acute OFP patients showed intermediate stress levels and did not differ significantly from either group.

**Figure 2:**
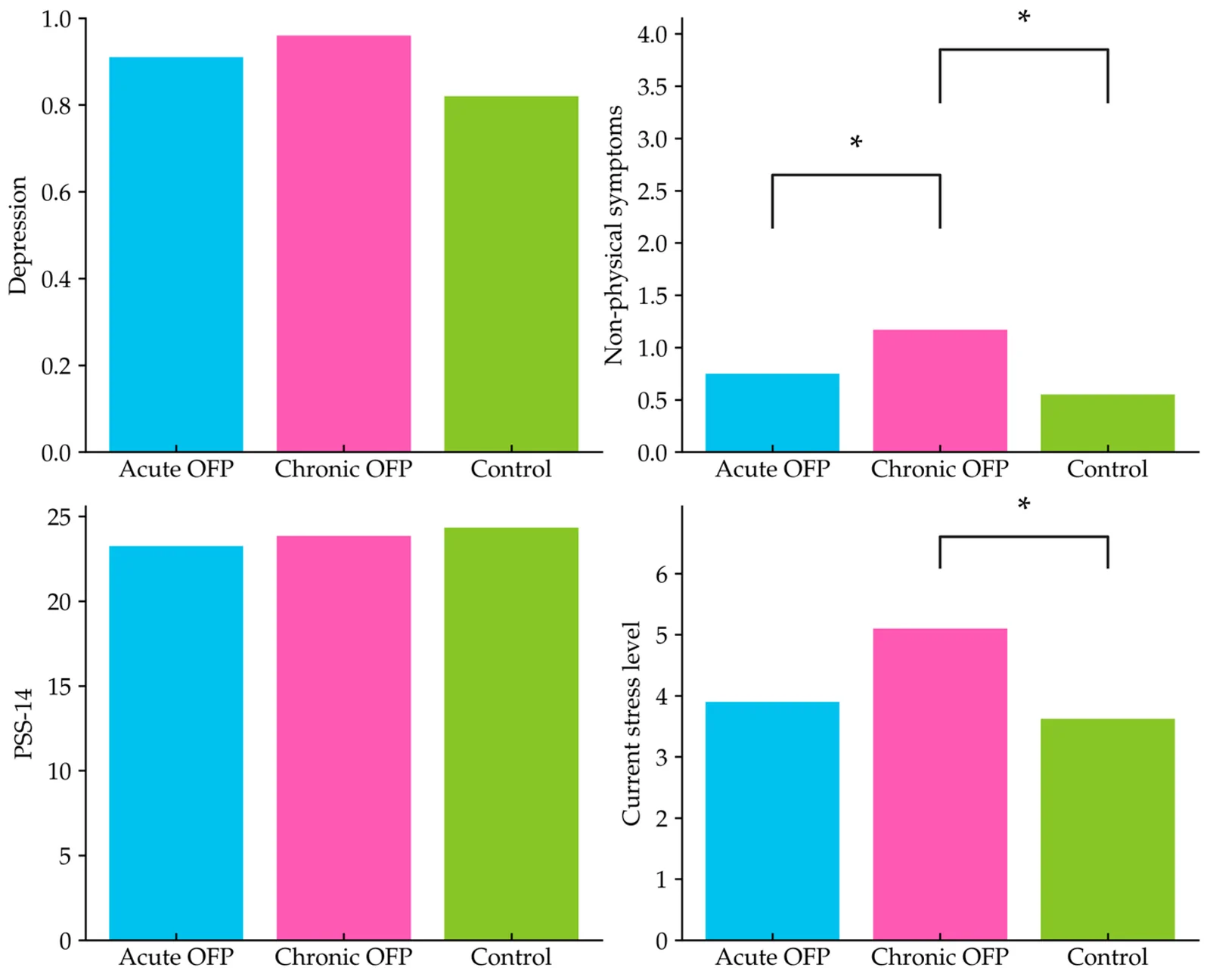
Psychological variables across study groups including perceived stress (PSS-14), current stress, depressive symptoms, and non-specific physical symptoms. Bars represent mean scores. Significant between-group differences are indicated. * *p* < 0.05.

No significant group differences were observed for perceived stress measured with the PSS-14 (*p* = 0.97) or depressive symptoms measured with the SCL-32-R (*p* = 0.27). Overall, participants across all groups reported moderate perceived stress and low-to-moderate levels of depressive symptoms (Figure 2).

### 3.5. General and Oral Health

Pain-free controls reported the most favorable ratings of general and oral health. In contrast, both pain groups reported lower levels of perceived health, particularly regarding oral health. A notable proportion of acute OFP patients rated their oral health as poor, whereas no chronic OFP patients reported poor oral health.

### 3.6. Correlation Analyses

Across all participants, sAA activity was weakly positively correlated with current pain intensity (r_s_ = 0.23, *p* < 0.007), but not with perceived stress, depressive symptoms, or non-specific physical symptoms. However, the pooled association between sAA activity and current pain intensity was considered descriptive because pain intensity differed systematically among the groups and controls reported no current pain by definition. In the group-specific analyses, a positive correlation was observed in the acute OFP group (r_s_ = 0.33, *p* = 0.03); however, this association did not meet the Bonferroni adjusted significance level (α-adjusted = 0.025). No association was observed in the chronic OFP group (r_s_ = 0.012 *p* = 0.938).

Within the acute OFP group depressive symptoms were strongly associated with non-specific physical symptoms (r_s_ = 0.72, *p* < 0.007), perceived stress (r_s_ = 0.52, *p* < 0.007). Perceived stress was also correlated with non-specific physical symptoms (r_s_ = 0.50, *p* < 0.007), and current stress (r_s_ = 0.48, *p* < 0.007).

Within the chronic OFP group, depressive symptoms were positively correlated with non-specific physical symptoms (r_s_ = 0.76, *p* < 0.007), perceived stress (r_s_ = 0.49, *p* < 0.007), and current stress (r_s_ = 0.44, *p* < 0.007).

Among controls, significant associations were observed between depressive symptoms perceived stress (r_s_ = 0.53, *p* < 0.007), and non-specific physical symptoms (r_s_ = 0.68, *p* < 0.007). Only correlations that remained significant after correction for multiple comparisons are reported.

## 4. Discussion

The primary hypotheses were only partly supported. Women with acute and chronic OFP showed partly distinct patterns of sAA activity and psychosocial symptoms. Participants with acute OFP had significantly higher sAA activity than pain-free controls and greater current pain intensity than participants with chronic OFP. However, sAA activity did not differ significantly between the acute and chronic OFP groups or between the chronic OFP group and controls. Participants with chronic OFP reported higher current stress and non-specific physical symptoms than controls, without a statistically significant elevation in sAA activity. However, perceived stress and depressive symptoms did not differ significantly between groups. Together, these findings may support the interpretation that sAA activity may be associated with short-term autonomic activation in the context of acute nociceptive stress, whereas no corresponding elevation in sAA activity was observed in the chronic OFP group despite the broader psychosocial burden associated with persistent orofacial pain. However, given the cross-sectional design and the single measurement of sAA, this interpretation remains tentative and does not establish a temporal or mechanistic relationship.

The higher sAA activity observed in acute OFP is consistent with previous studies showing that sAA responds rapidly to acute psychological and nociceptive stress through activation of the sympathetic-adrenomedullary system [18,24,27,28,35]. Patients with acute OFP also reported the highest current pain intensity. Although sAA activity was weakly positively correlated with current pain intensity across the entire study population, no significant association was observed within either the acute or chronic OFP group. The pooled correlation may therefore have been influenced by between-group differences, particularly because controls reported no current pain, and should not be interpreted as evidence that sAA tracks pain intensity at the individual level.

Since saliva samples were collected during ongoing pain, the elevated sAA activity may reflect an acute autonomic response to nociceptive stimulation and immediate distress associated with acute odontogenic pain [24,27,28]. However, the cross-sectional design and single sAA measurement do not allow the observed elevation to be attributed specifically to pain-related autonomic activation. Alternative explanations may include differences in pain intensity, age differences between groups, inflammatory characteristics of acute odontogenic conditions, analgesic or other medication use, and saliva-sampling conditions.

In contrast, patients with chronic OFP did not show significantly elevated sAA activity compared with pain-free controls. Despite the absence of a clear increase in sAA activity, patients with chronic OFP reported higher levels of current stress and non-specific physical symptoms, indicating a greater psychosocial and somatic burden. Previous research suggests that chronic pain may be associated with altered autonomic regulation and dysregulation of stress systems rather than continuous sympathetic activation [36,37]. Chronic OFP, including TMD, is considered a multifactorial condition in which biological, psychological, and social factors interact and contribute to pain persistence and symptom severity [10,11,38]. Stress regulation in chronic pain may therefore be more complex and heterogeneous than can be captured by a single measurement of sAA. Long-term pain exposure may also contribute to altered or attenuated autonomic responses over time. These mechanisms could partly explain why patients with chronic OFP exhibited greater current stress and non-specific physical symptoms without a corresponding elevation in sAA activity [13].

Although no significant differences were observed for depressive symptoms or perceived stress measured with the PSS-14, patients with chronic OFP reported significantly higher levels of non-specific physical symptoms and current stress compared with controls. The discrepancy between current stress and perceived stress over a longer time frame may reflect differences in the constructs and reference periods captured by the two measures. Current stress represents an immediate state, whereas the PSS-14 assesses perceived stress over a broader period. Strong correlations between depressive symptoms, perceived stress, and non-specific physical symptoms were also observed, particularly in chronic OFP group. These findings are in line with previous studies showing associations between chronic TMD pain and psychosocial symptoms [8,9,10].

Taken together with the absence of significantly elevated sAA activity in the chronic OFP group, these findings suggest that sAA may be more indicative of acute autonomic activation than of the broader psychosocial burden associated with persistent pain. A single sAA measurement may capture a transient sympathetic response at the time of saliva collection but cannot provide a comprehensive measure of long-term stress exposure, psychological distress, somatic symptom burden, or dysregulation across multiple stress-response systems. Chronic OFP should therefore not be reduced to the presence or absence of an altered autonomic biomarker. Rather, its biopsychosocial complexity requires biomarkers such as sAA to be interpreted together with clinical pain characteristics and psychological and somatic measures.

Several limitations should be considered when interpreting the present findings. First, this was an observational cross-sectional study, which precludes conclusions regarding causality, temporal changes, or the development of autonomic adaptation or dysregulation over time. Second, the acute and chronic OFP groups differed not only in pain duration but also in pain etiology, anatomical origin, pathophysiology, clinical setting, age, and likely inflammatory and nociceptive mechanisms. Consequently, the observed differences cannot be attributed to pain chronicity alone. Third, the relatively modest sample size limits the precision of the estimates and the statistical power of the group-specific correlation analyses. The weak pooled correlation between sAA and current pain intensity, together with the absence of significant within-group associations, further limits conclusions regarding an individual relationship between pain intensity and sAA. Fourth, saliva samples were collected at a single time point, which precluded assessment of intra-individual variation and circadian patterns in sAA activity. Although recorded sampling times did not differ significantly between groups, residual diurnal variation cannot be excluded. In addition, salivary flow rate was not measured and could therefore not be accounted for in the analyses, despite its potential influence on salivary biomarker levels [20]. Analgesic use was more frequently reported in the pain groups, particularly in the acute OFP group, and medication use may have influenced sAA activity. Moreover, lifestyle and hormonal factors, including caffeine consumption, smoking, oral contraceptive use, and hormonal treatment, were not fully controlled analytically. Information regarding previous treatment for orofacial pain and formal psychiatric diagnoses was also not systematically collected, and residual confounding related to medication use, psychological comorbidity, or other stress-related factors cannot be excluded. Finally, because only women were included, the findings cannot be generalized to the general population.

Despite these limitations, the present study has several important strengths. The inclusion of both acute and chronic OFP groups, together with pain-free controls, enabled direct comparison of distinct pain conditions within the same study framework. Chronic OFP was diagnosed using standardized DC/TMD criteria, and validated instruments were used to assess pain and psychosocial variables. In addition, saliva collection and biochemical analyses followed standardized procedures, and the combined assessment of a biological marker together with clinical pain and psychosocial measures provides a broader perspective on the multidimensional nature OFP. Future studies should include larger and more diverse samples, more comparable pain groups, repeated saliva sampling, assessment of salivary flow rate, and more detailed evaluation of oral status, psychiatric comorbidity, medication use, and previous pain treatment to further clarify the role of sAA in acute and chronic orofacial pain. Longitudinal studies could clarify whether sAA activity changes as acute OFP resolves or as chronic OFP responds to treatment.

From a clinical perspective, the present findings suggest that acute and chronic OFP may require partly different approaches to assessment. In acute OFP, higher sAA activity than in controls may, together with higher pain intensity may reflect short-term autonomic activation during ongoing nociceptive stress. In contrast, chronic OFP appears to be more closely linked to psychosocial symptom burden than to altered sAA activity. This supports the clinical relevance of assessing psychological and somatic symptoms alongside pain characteristics. Although sAA cannot currently be considered a standalone clinical biomarker, the present findings support a multidimensional approach to orofacial pain assessment in which biological, clinical, and psychosocial factors are considered together.

## 5. Conclusions

In conclusion, the primary hypotheses were only partly supported. These findings suggest that acute and chronic OFP are associated with partly different stress-related profiles. Women with acute OFP had higher sAA activity than pain-free controls, whereas women with chronic OFP reported higher current stress and non-specific physical symptoms without corresponding increase in sAA activity. However, perceived stress and depressive symptoms did not differ significantly between groups. This may indicate that sAA primarily reflects short-term autonomic activation in response to acute nociceptive stress rather than the more complex biopsychosocial processes underlying persistent pain; however, the cross-sectional design does not allow this mechanism to be established. From a clinical perspective, the results support the importance of integrating biological, clinical and psychosocial dimensions in the assessment of OFP. Further longitudinal studies are needed to clarify how salivary biomarkers relate to pain persistence, stress regulation, and treatment response over time.

## Figures and Tables

**Table 1:** Demographic and socioeconomic characteristics of female participants with acute and chronic orofacial pain (OFP) and healthy controls. Data are presented as number (*n*) of patients with percentage (%) unless otherwise specified.

	Acute OFP (*n* = 47)	Chronic OFP (*n* = 47)	Controls (*n* = 50)
**Age (Mean ± SD) years**	46.1 ± 13.9	36.2 ± 13.6	42.4 ± 14.1
**Education**			
None	2 (4)	0 (0)	1 (2)
Elementary school	9 (19)	2 (4)	4 (8)
High school education	24 (51)	18 (38)	28 (56)
University degree	10 (21)	24 (51)	13 (26)
Others	2 (4)	3 (6)	4 (8)
**Employment status**			
Gainful employment	30 (64)	27 (57)	35 (70)
Student	4 (9)	16 (34)	8 (16)
Housewife	0 (0)	0 (0)	0 (0)
Unemployed	6 (13)	0 (0)	1 (2)
Early pension	1 (2)	1 (2)	0 (0)
Sick leave	4 (9)	3 (6)	3 (6)
Retirement pension	2 (4)	0 (0)	2 (4)
Not given	0 (0)	0 (0)	1 (2)
**Marital status**			
Married/Partner	24 (51)	24 (51)	34 (68)
Single	17 (36)	19 (40)	13 (26)
Divorced	4 (9)	3 (6)	3 (6)
Widow	2 (4)	1 (2)	0 (0)

**Table 2:** Median (IQR) pain intensity according to NRS (0–10) and frequency of analgesic use presented as number (*n*) of patients with percentage among female participants with acute and chronic orofacial pain (OFP) and controls.

	Acute OFP (*n* = 47)	Chronic OFP (*n* = 47)	Controls (*n* = 50)
**Pain Intensity median (IQR)**			
Current pain	7 (3)	5 (3)	0 (0)
Worst pain	6 (3)	7 (2)	0 (0)
Average Pain	6 (3)	6 (2)	0 (0)
**Frequency *n* (%) of analgetic use**			
Never	3 (6)	5 (11)	25 (50)
1–2 times per month	10 (21)	13 (28)	13 (26)
1 time per week	3 (6)	12 (26)	4 (8)
>1 times per week	18 (38)	13 (28)	6 (12)
Daily	12 (26)	4 (9)	1 (2)
Not given	1 (2)	0 (0)	1 (2)

“Current pain” refers to the pain experienced at the time of assessment. Pain was self-reported using a numeric rating scale (NRS) from 0 (no pain) to 10 (worst pain imaginable). Data are presented as number (*n*) of patients with percentage (%) unless otherwise specified.

## Data Availability

The data presented in this study are available from the corresponding author upon reasonable request. The data are not publicly available because they contain sensitive personal information.

## Abbreviations

The following abbreviations are used in this manuscript:

DC/TMD	Diagnostic Criteria for Temporomandibular Disorders
NRS	Numeric Rating Scale
OFP	Orofacial Pain
PSS-14	Perceived Stress Scale 14-item
sAA	Salivary Alpha-Amylase
SCL-32-R	Symptom Checklist-90-Revised, short version
TMD	Temporomandibular Disorders
